# Optimized volume models of earthquake-triggered landslides

**DOI:** 10.1038/srep29797

**Published:** 2016-07-12

**Authors:** Chong Xu, Xiwei Xu, Lingling Shen, Qi Yao, Xibin Tan, Wenjun Kang, Siyuan Ma, Xiyan Wu, Juntao Cai, Mingxing Gao, Kang Li

**Affiliations:** 1 Key Laboratory of Active Tectonics and Volcano, Institute of Geology, China Earthquake Administration, Beijing, 100029, China; 2 Beijing Meteorological Information Center, Beijing Meteorological Service, Beijing, 100089, China; 3 China Earthquake Networks Center, Beijing, 100045, China; 4 Earth Observatory of Singapore, Nanyang Technological University, Singapore, 639798, Singapore

## Abstract

In this study, we proposed three optimized models for calculating the total volume of landslides triggered by the 2008 Wenchuan, China Mw 7.9 earthquake. First, we calculated the volume of each deposit of 1,415 landslides triggered by the quake based on pre- and post-quake DEMs in 20 m resolution. The samples were used to fit the conventional landslide “volume-area” power law relationship and the 3 optimized models we proposed, respectively. Two data fitting methods, i.e. log-transformed-based linear and original data-based nonlinear least square, were employed to the 4 models. Results show that original data-based nonlinear least square combining with an optimized model considering length, width, height, lithology, slope, peak ground acceleration, and slope aspect shows the best performance. This model was subsequently applied to the database of landslides triggered by the quake except for two largest ones with known volumes. It indicates that the total volume of the 196,007 landslides is about 1.2 × 10^10^ m^3^ in deposit materials and 1 × 10^10^ m^3^ in source areas, respectively. The result from the relationship of quake magnitude and entire landslide volume related to individual earthquake is much less than that from this study, which reminds us the necessity to update the power-law relationship.

The volume of landslides triggered by major earthquakes is an important parameter for assessment of geohazards, study of post-seismic debris flow^1^ and impact of landslides on geomorphologic evolution^2^,^3^,^4^,^5^. However, this value is generally difficult to determine due to its three-dimensional nature and the hidden sliding surfaces underground. Keefer^6^ correlated the earthquake magnitude with total volume of quake-triggered landslides based on 15 historical events and fitted a power law relationship. This relationship has been subsequently applied in several case studies^7^,^8^,^9^. However, it should be noted that the volumes of landslides from the 15 earthquakes collected by Keefer^6^ may have large errors due to the use of various methods for volume calculation. For example, based on the power law relationship of the volume and area of individual seismic landslides proposed by Simonett (1967)^10^, Adams^11^ respectively calculated the volumes of landslides triggered by 3 earthquakes, which are the June 17, 1929 Buller M 7.6, the March 9, 1929 Arthur’s Pass M 6.9, and the May 23, 1968 Inangahua M 7.1 earthquakes in New Zealand. However, he^11^ did not introduce inventories of landslides related to these 3 events. It can be inferred that there were significant errors in those volume values because the underdeveloped remote sensing and GIS technologies at that time which can influence the quality of landslide inventories. Subsequently, Pearce and O’Loughlin^12^ estimated that the percentage of the coverage area of the landslides triggered by the 1929 Buller M 7.6 event was about 4%, which was based on several previous studies^10^,^13^,^14^,^15^,^16^, and assumed that the average depth of the landslides was about 5 m. They calculated the total volume of the landslides triggered by the quake in the 5000-km^2^ quake-affected area to be 5000 km^2^ × 4% × 5 m, or 1 × 10^9^ m^3^. Although it is comparable with the result 1.3 × 10^9^ m^3^ from Adams^11^, several important indexes, such as landslide area percentage and average depth of landslides, were from expert knowledge which was subjective. Another example is the August 15, 1950 India’s Assam M 8.6 earthquake. Mathur^17^ found that landslides triggered by this event covered 15,000 km^2^ and at least 5.0 × 10^10^ m^3^ materials were dislodged^18^, which is 4.7 × 10^10^ m^3^ cited by Keefer^6^. However, only aerial reconnaissance and expert knowledge were used in this estimation, which could generate significant errors in the results.

In recent years, with the application of well-developed remote sensing and GIS technologies, quite a few detailed inventories of quake-induced landslides have been successively constructed. In terms of the landslide area-volume scaling relationships, these detailed inventories were used to calculate total volume of coseismic landslides by major events, such as the 2008 Wenchuan, China^3^,^4^,^5^, 2010 Yushu, China^19^, 2010 Haiti^8^, 2013 Lushan, China^9^ earthquakes. Nevertheless, due to various landslide depths, the resultant volume values are highly variable even though with comparable landslide areas. For example, Larsen *et al*.^20^ fitted two power law relationships of the landslide volume and area for shallow, soil-based slides and bedrock landslides and found that the failure of bedrock has a deeper scar area, and hence a larger volume and power exponent. In summary, through retrospective of the previous studies, we find that calculation of the landslide volume still relies on simple landslide “volume–area” power law relationships, which have significant errors.

In this work, based on the database of 196,007 landslides triggered by the 2008 Wenchuan, China Mw 7.9 earthquake^21^, we added several attributes to the model on the landslide area-volume scaling relationship aforementioned, such as landslide area, length, width, height, lithology, slope, peak ground acceleration, and slope aspect, to improve the estimation of landslide volumes. For a whole landslide body, the length represents the maximum horizontal length of the landslide. The width means average value, which is calculated by dividing landslide area by landslide length. The height refers to the difference between the highest and the lowest elevation of a landslide body. Pre- and post-quake DEMs (20 m resolution) were used to calculate deposit volumes of 1,415 individual landslides, which were used as samples to fit landslide volume models. Base on the traditional power law relationship, we proposed three optimized models and employed log-transformed-based linear least square and original data-based nonlinear least square fitting methods to regress coefficients of these models. The 1,415 samples were used to validate the traditional model and the three optimized models. The results show that the original data-based nonlinear least square regression considering landslide geometry parameters, topography, lithology, and PGA has the lowest standard deviation and presents the best performance. This optimized model is subsequently used to the database of all landslides triggered by the Wenchuan earthquake, yielding the total volume of landslides triggered by the quake about 1.2 × 10^10^ m^3^ for deposit materials and 1 × 10^10^ m^3^ in scar areas.

## Data and Methods

### Volume samples of individual landslides

**Figure 1:**
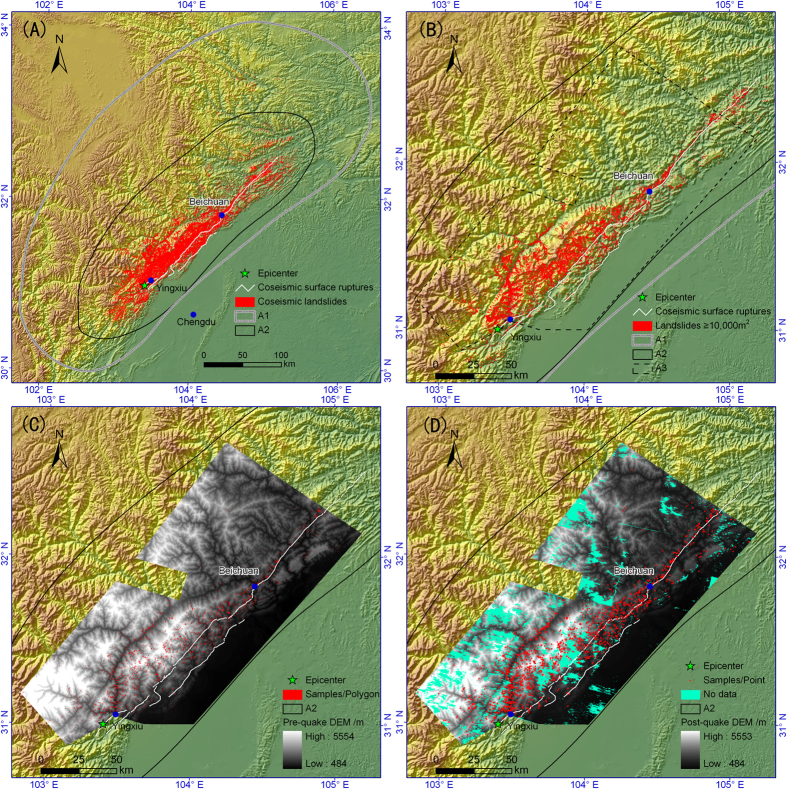
Distribution maps of landslides triggered by the 2008 Wenchuan earthquake and landslide volume samples. (**A**) Distribution of 197,481 landslides^21^. (**B**) Distribution of 25,580 individual landslides with area larger than 10,000 m^2^. (**C**) 1,415 landslide samples (denoted by polygons) on pre-quake DEM (20 m). (**D**) The samples (denoted by points) and areas without data on post-quake DEM (20 m). A1, the approximately distribution elliptical area of landslides larger than 110,000 km^2^; A2, the 44,000-km^2^ area with relatively higher landslide abundance; A3, the coverage area of pre- and post-quake DEMs. The colorful hillshade backgrounds on the panels A-D are from SRTM DEM^23^ (90 m) based on the ArcGIS 9.2 Platform (http://www.esri.com/apps/products). The pre- and post-quake grey DEMs on the panels C and D are from SPOT 5 stereoscopic pairs of images. All the four figures were also generated using the ArcGIS 9.2.

The 2008 Wenchuan event triggered at least 197,481 landslides, with a total coverage area 1,160 km^2^ and an approximately distribution elliptical area larger than 110,000 km^2^ (A1 in Fig. 1A). The smallest landslide only covers 30 m^2^. Of the big dataset, 196,007 landslides with a total coverage area 1150.8 km^2^ are located in a 44,000-km^2^ area^21^ with relatively higher landslide abundance (A2 in Fig. 1A), including 25,580 individual landslides with planar area larger than 10,000 m^2^ (Fig. 1B). The quake ruptured two main faults and generated two surface ruptures, which are 240 km and 72 km long^22^. The pattern of the coseismic landslides shows a consistent distribution with the coseismic ruptures. We collected SPOT 5 stereoscopic pairs of image-derived pre- and post-quake DEMs (Fig. 1C,D) with high resolution (20 m) in a 19,969-km^2^ irregular polygon (A3 in Fig. 1A), which is totally enclosed in the landslide dense area (A2 in Fig. 1A). The quality of pre-quake DEM is quite satisfactory (Fig. 1C) whereas the post-quake DEM shows about 2,483-km^2^-covered patches without data (Fig. 1D), which was probably due to the impact of clouds. The areas without data were filled by the linear interpolation method and then the pre-and post-quake DEMs were employed to derive DEM differentials, which were used for calculating individual landslide volumes of deposit areas and scar areas.

The correlation curve between landslide area and frequency related to the Wenchuan quake^21^ shows a downward bend near 10,000 m^2^ of the landslide area, which implies some possible missing of small-scale landslides. In addition, the error of landslide volume increases with the decreasing landslide scale. Therefore, 170,427 landslides with area less than 10,000 m^2^ were compulsory excluded and the remaining 25,580 large landslides were used in subsequent analysis. The landslides located out of the DEM coverage were also excluded from the sample set, consequently 19,279 landslides left. The differential of the pre- and post-quake DEMs were derived by the Spatial Analyst module of ArcGIS, and thus the net deficit and increment related to each landslide sample was obtained. It should be noted that the net deficit and increment are still not the volumes of landslide deposits and landslide scar areas, because they generally do not have clear boundaries, which means that the bottom of the source and accumulation area of a landslide are generally not exposed or some of the landslide materials stay in the source region. Especially for deep-seated landslides with short runout distances, the source area and accumulation area significantly coincide with each other. Although we collected many cross-sectional diagrams of the landslides, the quality of them was not always satisfactory. Therefore, we set 10% based on field observations and analysis of remote sensing images as the percentage of the failure materials that stays in the scar region to the volume of the scar area for most of the landslides have no cross-sectional diagrams. However, it should be noted that the limitation of the value 10% is somewhat subjective due to the inaccessible sliding surfaces covered by landslide deposits and the three-dimensional nature of the landslide deposits. Then, the volume of deposits and accumulation materials of each landslide sample were calculated based on the net deficit and increment from DEM differentials.

The three following criteria were used to ensure the quality of the samples of landslide volume: (1) The occurrence of a landslide, which experienced a failure, runout, and accumulation process, means that intact and low porosity source materials might be transformed into loose and higher porosity deposits, and thus leading to the volume of the accumulation deposit larger than the source material. A few case studies show the expansion rate of a landslide (the percentage of increment volume to source volume) is about 20%^24^,^25^. Therefore, we selected 0–50% as the expansion rate threshold for slope failures. Those samples with abnormally large or small values of expansion rates were compulsory excluded. (2) We selected the percentage of volume of accumulation materials for a landslide from DEM differential to estimate volume based on a “volume-area” power law relationship V = 0.106 × A^1.388^ from Parker *et al*.^4^ ranging 20–500%, thus excluding sample outliers covered by low quality post-quake DEM, which can improve the quality of the samples. (3) Each landslide was carefully checked with post-quake DEM to exclude those samples covered by interpolation areas of certain proportions (about larger than 10%). With constraints of the above qualifications, 1,417 samples left. According to the power law relationship between landslide area and frequency related to the Wenchuan quake^21^, the Daguangbao and Wenjiagou landslides, the two largest landslides triggered by the quake, present two outliers due to their extremely large volumes^21^. Therefore, they were compulsory excluded from the sample set. Finally, 1,415 landslides, with reliable volume values, were used as samples for subsequent model testing. The total area of these landslides is 41,944,490 m^2^ and total volume of the landslide deposits is about 525,959,640 m^3^. The “volume-area” power law relationship V = 0.106 × A^1.388^ was also used to calculate the volume of the samples, which indicates a much smaller total volume 303,710,242 m^3^.

### Models for landslide volume

The power law relationships between the landslide volume and area are often employed in calculating volumes of landslides on regional scales because the parameter area of landslides is relatively easy to determine. However, landslides with comparable areas probably have significant volume differences due to highly variable landslide types, thickness, and materials. This may result in an obvious difference between the real volume and that from the power relationship for a landslide. Therefore, in this study, more factors including landslide area (A), length (L), width (W), height (H), lithology (Lith), slope (Slp), peak ground acceleration (PGA), and slope aspect (Asp) were introduced into calculation of regional landslide volumes. The geometric parameters of a landslide, such as the area, length, width, and height definitely affect its volume because the volume is a three-dimensional space decided by these factors. Lithology can also play an important role in landslide size. Previous work shows that soil landslides occurring in loose, weathered layers usually have a shallower scar area than bedrock landslides^20^. Slope angle not only strongly controls the occurrence of a landslide, but also affects its volume through affecting the failure mode, runout distance, and accumulation area of the landslide. PGA is a reflection of the local earthquake energy suffered by the landslide, which affects the initial speed, runout distance, and accumulation area for the slide. Slope aspect may affect the correlation between landslide volume and area because of the interaction between the slope aspect and the directions of block movement and seismic wave propagation^8^,^26^.

Therefore, the function of landslide volume (V) can be expressed in a general fashion as:


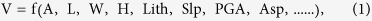


where V is the volume of a landslide, A is landslide area, L is the largest length of the slide or the farthest runout distance of landslide materials, W is the average width of the landslide, which is obtained by landslide area divided by the length, H is landslide height, which is the elevation differential between the peak elevation of the scar area and the lowest elevation of the deposit area, Lith represents the local lithology of the landslide, Slp is slope angle, PGA is peak ground acceleration, and Asp is slope aspect.

The conventional power law relationships between the landslide volume and area is


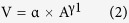


which can be regarded as the most simple form of equation (1). The power law relationship between landslide volume and area indicate that there may be similar relationships between landslide volume and other factors, such as landslide length, width, height, lithology, slope, PGA, and slope aspect. Therefore, this work considered more parameters and used the following forms of the general function equation (1):


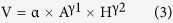



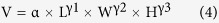



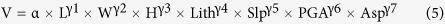


where α, γ1, …… , and γ7 are coefficients, and definitions of other parameters have been presented above. The values of Lith, Slp, PGA, and Asp are from a previous study^27^. The coefficients in equations (2, 3, 4, 5) can be fitted based on the sample set mentioned earlier on the basis of the nonlinear least square method.

The nonlinear equations (2, 3, 4) can be transformed into linear ones by a logarithmic tool. For example, V = α × A^γ1^ can be transformed into ln(V) = γ1 × ln(A) + ln (α), which can be rewritten as a binary linear equation Y = a × X + b, here Y = ln(V), a = γ1, X = ln (A); b = ln (α). In this way, equations (2, 3, 4, 5) were converted into.


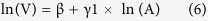



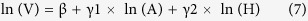



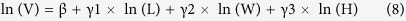



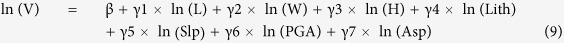


Thus, the nonlinear equations (2, 3, 4, 5) were converted into linear equations (6–9) and log-transformed data can be used to fit equations (6–9) based on the linear least square method. The advantage of this linear tool is easier than the nonlinear method, whereas its disadvantages are bigger errors from high-order data, such as larger-scale landslides. On other hand, the nonlinear least square method on the basis of original data considers the more significant influence of high-order data, which will have relatively smaller errors. Therefore, this work employed both methods and compared their results.

## Results and analysis

### Correlations between landslide volume and area, length, width, and height

**Figure 2:**
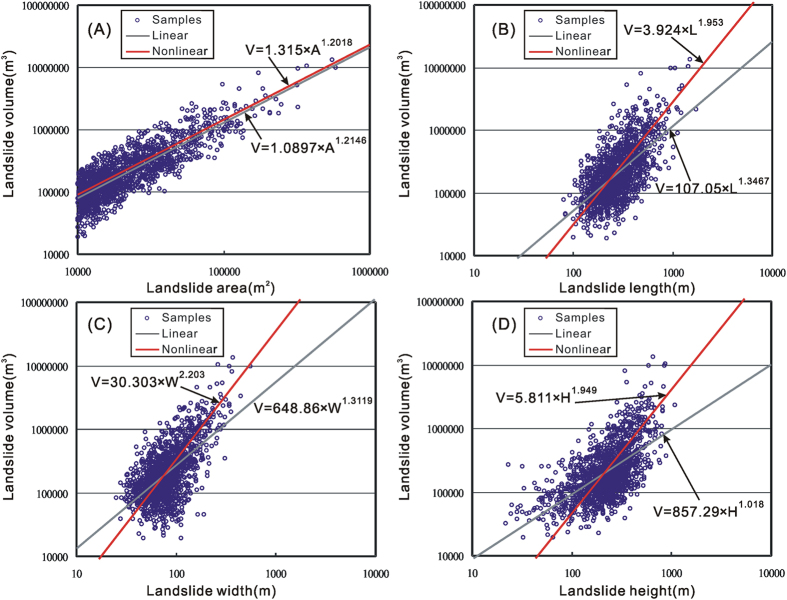
Correlations between landslide volume and area (**A**), length (**B**), width (**C**) and height (**D**). Regression analysis.

This work has obtained correlations between volume and area (A), length (L), width (W), and height (H) of the aforementioned 1,415 samples. They are projected on four panels (Fig. 2). Regression curves on the basis of the two data fitting methods, i.e. log-transformed linear least square and original data-based nonlinear least square were derived for each correlation (Fig. 2). The resultant curves from the linear least method shows low-quality regression because the method did not work well for data in multiply orders. In other words, significant errors occurred. However, the nonlinear method solved this problem properly (Fig. 2). The data points of larger landslides are closer to the red lines (nonlinear fitted method) than that to the gray lines (linear fitted method) (Fig. 2). The coefficients of determinations (R^2^) of the linear correlations between log-transformed landslide volume and area, length, width, and height are 0.7108, 0.3941, 0.3838, and 0.532, respectively, whereas the R^2^ values of the nonlinear correlations on the basis of original data are 0.777, 0.405, 0.532, and 0.267, respectively, which indicates that the correlation between landslide volume and area is better than those correlations between landslide volume and other geometric parameters. This is perhaps because one of the aforementioned constraints for sample selection is the 20–500% for the percentage of landslide volume from DEM differential to estimated volume on the basis of the “volume–area” power law relationship V = 0.106 × A^1.388^. The relative low R^2^ values of correlations between landslide volume and length, width, and height are probably because landslides with comparable volumes are of various types and have considerable differences in lengths, widths, and heights. On the other hand, it also shows that the volume of landslides may be affected by more parameters in addition to geometry parameters.

The two fitting methods aforementioned were employed again to regress the four models for calculations of landslide volume, which include one conventional model (equation 2) and three optimized models (equations 3, 4, 5). Table 1 shows the result of the linear fitting method for these models. Coefficients of determinations (R^2^) from types 1 to 4 (Table 1) increase from 0.7108 to 0.7546 and standard deviations for log-transformed volume (SD-LT) decrease from 0.517 to 0.4772, which indicates that the increasing reliability of the four models. In order to check whether the linear least square method can solve the nonlinear problem, we compared the real volumes of samples with the regression volume results derived from each model. Then we calculated the standard deviations of landslide volume (SD-V) for each model as well as total volume of the landslide samples and percentage of regression for the total volume to the real total volume of the 1,415 landslides (Table 1). It indicates that the standard deviations of landslide volume show an irregular variation tendency compared with R^2^ and standard deviations for log-transformed volume. Thus it shows that the log-transformed linear least square method is not suitable for nonlinear questions even though the percentage of the whole regression volume to real total volume of the samples looks rather high (87.23–88.07%).

**Figure 3:**
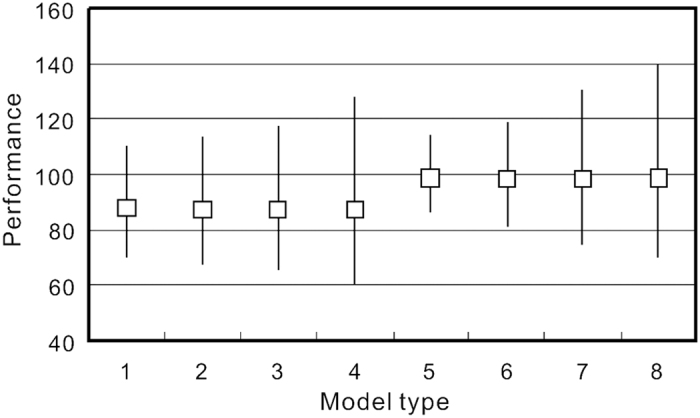
Performances and uncertainties of each model in the Tables 1 and 2. The performance means the percentage of calculated total volume to real total volume of the 1,415 samples. Squares denote the regressed values. Upper and lower vertical lines denote the uncertainties of volume% considering of ±1σ on γ1.

Next, the nonlinear equations were regressed by the nonlinear least square method through iteration. Original data were directly used in modeling without any data transformation but the initial values of every coefficient were set for the iteration. In order to avoid iteration failure caused by big gaps between the initial values and actual values, the coefficients in Table 1 were employed as the initial values for equations (6–9). Results in Table 2 show significant differences to the linear method in Table 1. Similar to Table 1, the orders of R^2^ generally increase from 0.777 to 0.787. However, standard deviations of the landslide volume generally decrease from 365,614 m^2^ to 358,79 m^2^, which is different from the cases in Table 1. Therefore, it shows the increasing reliability of the four models (Table 2) on the basis of the original data-based nonlinear method. The percentage of regressed total volume of the samples to the real one does not show similar tendency, which is probably because the volumes of most landslides are less than real values. The optimal model in this study aims at the closest fitting between the regression value and true value for every landslide, rather than only the accuracy of the total landslide volume. Furthermore, the total landslide volumes of the four models are very close to the true values, with a percentage range 97.98–98.91% (Fig. 3). Therefore, the type 8 (equation 9) is the best model by considering values close enough between regression and reality for individual landslides.

There are considerable differences between the results from linear (Table 1) and nonlinear (Table 2) methods. The coefficients of determination increase from 0.7108 to 0.7546 for the linear method, while ranging in “0.777–0.787” for the nonlinear method, standard deviations of landslide volume decrease from “374599–409812 m^3^” to “358479–365614 m^3^”, and the percentage of total regressed volume to total real volume of the 1415 landslides increase from “87.23–88.07%” to “97.98–98.91%”.

For the nonlinear equations of regional landslide volume calculation, the original data-based nonlinear least square shows much better performance than the log-transformed linear least square. However, such a difference in performance has not been concerned in previous studies. On power law relationships between the landslide volume and area, apparently linear methods were used in several studies^4^,^6^,^28^. Other studies^20^,^29^,^30^,^31^,^32^,^33^,^34^ did not present their fitted methods. It is estimated that the log-transformed linear square method has the most possibility as it can greatly simplify the calculation and consume less computing sources. Guzzetti *et al*. applied least square linear fit, robust resistant regression, robust linear fit, and least square nonlinear fit in their work^35^ to fit the log-transformed data. Although various data fitting methods were applied, data in their study has already been log-transformed, thus the nonlinear equation was converted into a linear one, yielding similar results on the basis of the four data fitting methods stated in this paper.

### Volume of landslides caused by the 2008 Wenchuan earthquake

This study used the power law relationship between landslide volume and area on the basis of the nonlinear fitting least square method, i.e. V = 1.315 × A^1.208^, which is different from that of Parker *et al*.^4^, i.e. V = 0.106 × A^1.388^. The coefficient *α* in this study is as large as 1.315, whereas another coefficient, exponent *γ* is 1.208, which is smaller than the latter. The volume (V) intersection point about the area (A) is 1,190,326 m^2^, which means only two landslides, the Daguangbao and Wenjiagou landslides, have areas larger than the intersection point value. The former curve shows slightly gentler than the latter in the log-transformed coordinate system. The significant difference in sample selection perhaps is more important than the fitting method. We assumed that both the 1,415 samples in this study and 41 samples measured by Parker *et al*.^4^ are accurate and objective and large bedrock landslides have relatively deeper scar areas, thus a larger value exponent γ^20^. Therefore, the difference is probably partly because the two aforementioned largest landslides were excluded from our sample set and the percentage of small-scale landslide in the total samples larger than the case in Parker *et al*.^4^. Although the sample set in this study is greater than 10,000 m^2^ in the landsliding area, they also show a similar power law relationship between the scale and frequency, which means the larger the area, the smaller the proportion of the landslides in the sample set. Conventionally, the 41 samples from Parker *et al*.^4^ are more likely characterized by large bedrock landslides in order to guarantee the accuracy. However, there are no specific and detailed descriptions on the 41 landslide samples^4^. The aforementioned optimized models in this study mainly focus on earthquake-triggered landslides and all samples are from the database of landslides triggered by the 2008 Wenchuan earthquake. However, these models undergoing some parameter changes are expected to suitable for nonseismic landslides. For example, as a trigger condition of landslides, PGA can be replaced by precipitation for regional rainfall landslides.

All the 196,007 landslides triggered by the 2008 Wenchuan earthquake in our database^21^ were assigned several attributes, including area, length, width, height, lithology, slope, peak ground acceleration, and slope aspect. The two largest landslides (Daguangbao and Wenjiagou landslides) triggered by the quake are outliers to the relationship between landsides area and accumulation frequency^21^ due to their extremely huge volumes. In addition, there are also monographic studies^36^,^37^ on accurate volumes of them, which are about 1 × 10^9^ m^3^ and 1 × 10^8^ m^3^, respectively. Therefore, the real volumes of the two landslides were employed rather than regression values. The volumes of each remaining 196,005 landslide were calculated on the basis of the equations in Tables 1 and 2. Table 3 shows the total volumes of the 196,005 landslides on the basis of the eight linear and nonlinear equations.

Finally, on the basis of the total volumes of the 196,005 landslides (Table 3) and the real volumes of the two largest landslides triggered by the Wenchuan quake, results of the 196,007 landslides are 1.1177 (0.9311–1.3478) × 10^10^ m^3^, 1.121 (0.9032–1.3987) × 10^10^ m^3^, 1.1502 (0.9037–1.4746) × 10^10^ m^3^, and 1.1651 (0.8559–1.6058) × 10^10^ m^3^ by considering the linear models (Table 1), respectively, whereas results of the nonlinear cases (Table 2) are 1.2487 (1.1145–1.4137) × 10^10^ m^3^, 1.2112 (1.0358–1.427) × 10^10^ m^3^, 1.2112 (0.9626–1.5408) × 10^10^ m^3^, and 1.1921 (0.9019–1.6074) × 10^10^ m^3^, respectively. Therefore, we considered 1.2 (0.9–1.6) × 10^10^ m^3^, mainly refers to the result from the nonlinear model 8, as the total volume of the 196,007 landslides triggered by the Wenchuan quake. It should be noted that the volume in the models are for deposit materials. The process of a landslide is intact and low porosity materials are converted into loose and higher porosity materials, thus the landslide expansion rate should be considered and the total volume of the scar areas of the landslides is about 1 (0.75–1.33) × 10^10^ m^3^ when the expansion rate is set to be 20%^24^,^25^. This result provides a useful supplement to previous studies^4^,^5^. According to the empirical formula between earthquake magnitude and total volume of associated landslides^6^, V = Mo/10^18.9(±0.13)^, the volume of landslides triggered by the Wenchuan Mw 7.9 quake (Mo = 8.97 × 10^27^ dyne-cm from www.globalcmt.org/CMTsearch.html) should be 0.113 × 10^10^ m^3^ (0.084–0.152 × 10^10^ m^3^), which only accounts for 9.42% (5.25–16.89%) and 11.3% (6.32–20.27%) of the new results of the total deposit and scar volumes from this work, respectively. It is not unusual that shallow earthquakes with comparable magnitudes could have large differences in the overall incidence and severity of landsliding. Local topography, geology, and nature of seismogenic faults also play important roles in coseismic landslide occurrence. The binary relationship between earthquake magnitudes and total volumes of coseismic landslides could have significant uncertainties. Therefore, it is necessarily to update the power law relationship based on more samples and model parameters.

## Conclusions

In this study, we proposed three optimized models for calculation of the landslide volume on the basis of a conventional power law relationship between the landslide volume and area. The conventional model and the three optimized models were used to calculate the volume of landslides triggered by the 2008 Wenchuan quake. The pre- and post-quake DEMs with 20 m resolution, derived from pre- and post-quake SPOT satellite images, were employed to prepare the landslide volume sample set. A total of 1,415 landslides were selected from all 196,007 landslides triggered by the Wenchuan quake. Two data fitting methods, i.e. log-transformed linear least square and original data-based nonlinear least square, were employed to fit the four equations between landslide volumes and associated geometry, topographic, geologic, and seismic parameters. The nonlinear models show better performance than the linear ones in the forms of higher coefficients of determination, lower standard deviations of landslide volume, as well as closer regression adduct volume of all landslides to actuality. The volumes of every landslide in the database were calculated except to the two largest landslides (Daguangbao and Wenjiagou cases) on the basis of the most optimal model which has been proved in this study, which is V = 0.114 × L^1.033^ × W^1.204^ × H^0.186^ × Lith^−0.021^ × Slp^0.173^ × PGA^0.643^ × Asp^0.187^. Combined with the actual volumes of the two largest landslides, the total volume of the 196,007 landslides triggered by the 2008 Wenchuan Mw 7.9 event is about 1.2 × 10^10^ m^3^ for accumulate materials, or 1 × 10^10^ m^3^ for scar areas. Results are expected to have positive significance to discover correlations between earthquake magnitude and volume of associated landslides, mitigation of debris flows and research of landscape evolution in the quake-affected area.

## Additional Information

**How to cite this article**: Xu, C. *et al*. Optimized volume models of earthquake-triggered landslides. *Sci. Rep.* **6**, 29797; doi: 10.1038/srep29797 (2016).

## Figures and Tables

**Table 1:** Four regression results for landslide volume on the basis of log-transformed linear method.

Type	ln(V) =	R^2^	SD-LT	SD-V(m^3^)	RV(m^3^)	Volume%	Uncertainty%
1	0.0859 + 1.2146 × ln (*A*)	0.7108	0.517	374599	463203533	88.07	70.37–110.29
2	0.0127 + 1.0524 × ln(*A*) + 0.3151 × ln (*H*)	0.735	0.495	391354	459808902	87.42	67.09–113.91
3	0.3527 + 0.612 × ln(*L*) + 1.2108 × ln (*W*) + 0.582 × ln (*H*)	0.7533	0.4777	405017	458797706	87.23	65.16–117.41
4	0.7929 + 0.5934 × ln(*L*) + 1.2144 × ln(*W*) + 0.5998 × ln (*H*) + 0.0051 × ln (*Lith*) −0.0296 × ln (*Slp*) −0.1131 × ln (*PGA*) −0.0496 × ln (*Asp*)	0.7546	0.4772	409812	458871654	87.24	59.98–127.8

Note: R^2^ is coefficient of determination. SD-LT is standard deviation of log-transformed volume. SD-V is standard deviation of landslide volume. RV is regression volume of the samples; volume % is percentage of regressed total volume of the samples to the real total volume; Uncertainty% is the range of volume% considering of ±1σ on γ1.

**Table 2:** Four regression results for landslide volume on the basis of original data-based nonlinear method.

Type	V=	R^2^	SD-V(m^3^)	RV(m^3^)	Volume%	Uncertainty%
5	1.3147 × A^1.2085^	0.777	365614	520235492	98.91	86.21–114.7
6	0.9105 × A^1.1693^ × H^0.1348^	0.779	364191	515352297	97.98	81.08–119.07
7	0.8919 × L^1.0493^ × W^1.2188^ × H^0.2223^	0.78	363428	516017809	98.11	74.39–130.36
8	0.1143 × L^1.0334^ × W^1.2036^ × H^0.1863^ × Lith^−0.0208^ × Slp^0.1725^ × PGA^0.6434^ × Asp^0.187^	0.787	358479	518046560	98.5	70.23–140.05

Note: The meanings of R^2^, SD-V, RV, volume%, and uncertainty% are same as Table 1.

**Table 3:** Total regression volumes of the 196,005 landslides on the basis of each model.

Model type	Volume (10^10^ m^3^)	±1σ on γ1	Volume range (10^10^ m^3^)
1	1.0077	±0.0206	0.8211–1.2378
2	1.011	±0.0244	0.7932–1.2887
3	1.0402	±0.049	0.7937–1.3646
4	1.0551	±0.0629	0.7459–1.4958
5	1.1387	±0.0131	1.0045–1.3037
6	1.1012	±0.0176	0.9258–1.317
7	1.1012	±0.0463	0.8526–1.4308
8	1.0821	±0.0571	0.7919–1.4974
